# Emerging Polymer-Based Nanosystem Strategies in the Delivery of Antifungal Drugs

**DOI:** 10.3390/pharmaceutics15071866

**Published:** 2023-07-01

**Authors:** Yuan Xin, Liang Quan, Hengtong Zhang, Qiang Ao

**Affiliations:** NMPA Key Laboratory for Quality Research and Control of Tissue Regenerative Biomaterial & Institute of Regulatory Science for Medical Device & National Engineering Research Center for Biomaterials, Sichuan University, Chengdu 610064, China; xinyuanscu@stu.scu.edu.cn (Y.X.); quanliang@stu.scu.edu.cn (L.Q.); zhanght_1226@163.com (H.Z.)

**Keywords:** antifungal, drug delivery, polymers, nanometer

## Abstract

Nanosystems-based antifungal agents have emerged as an effective strategy to address issues related to drug resistance, drug release, and toxicity. Among the diverse materials employed for antifungal drug delivery, polymers, including polysaccharides, proteins, and polyesters, have gained significant attention due to their versatility. Considering the complex nature of fungal infections and their varying sites, it is crucial for researchers to carefully select appropriate polymers based on specific scenarios when designing antifungal agent delivery nanosystems. This review provides an overview of the various types of nanoparticles used in antifungal drug delivery systems, with a particular emphasis on the types of polymers used. The review focuses on the application of drug delivery systems and the release behavior of these systems. Furthermore, the review summarizes the critical physical properties and relevant information utilized in antifungal polymer nanomedicine delivery systems and briefly discusses the application prospects of these systems.

## 1. Introduction

Fungi comprise millions of species on earth, with approximately 400 causing human diseases [1,2,3]. These infections can affect various parts of the body, including the skin, nails, soft tissues, lungs, blood, and brain [4,5,6,7,8]. Pathogenic fungi cause approximately 100 million infections worldwide every year, resulting in more than 60,000 deaths [9,10]. Unlike bacterial pathogens, fungal infections are mainly treated with five classes of drugs, namely, azoles (for systemic and superficial fungal infections) [11,12], polyenes (for severe systemic fungal infections) [13], echinocandins (for intractable fungal infections) [14,15], allylamines (which inhibit squalene epoxidase activity and disrupt the ergosterol synthesis pathway) [16], and antimetabolites (which inhibit fungal RNA and DNA synthesis) [17].

Although currently available antifungal drugs have demonstrated high efficacy in treating superficial and invasive fungal infections, their usage is often associated with side effects and limitations. Allylamines, for example, are mostly used for treating superficial fungal infections [18], while resistance to azoles is becoming increasingly prevalent [19,20]. Additionally, polyenes may cause infusion-related reactions [21]. Furthermore, these drugs may exhibit higher levels of toxicity when administered in vivo, which is a common challenge associated with antifungal drug therapies.

In addition to exploring new antifungal drug species, researchers are investigating the potential applications of nanotechnology in antifungal drug delivery [22]. Nanosystems-based drug delivery can achieve high local drug concentrations at the targeted site, thereby facilitating antifungal effects through various mechanisms, such as interfering with fungal membrane integrity through charge interactions, promoting the formation of reactive oxygen species (ROS), and altering the permeability of fungal cell membranes [23,24,25]. As a result, drug delivery nanosystems have emerged as an ideal mode of drug delivery [26].

Nanosystems typically exhibit submicrometer-sized structures, and their behavior can be influenced by several factors, such as the mode of administration, blood circulation time, and human immunity. Furthermore, the physicochemical properties of nano-drug delivery systems play a critical role in the release behavior of drugs. Researchers have conducted numerous studies on the matrix of antifungal drug delivery nanosystems, which include liposomes and lipoid vesicles [27], microemulsions, polymers [28], dendrimers, and inorganic materials (e.g., silicon-based [29], carbon materials, and metals [30]). These structures have been explored for their potential to enhance drug delivery efficiency, specificity, and safety.

Currently, there are several reviews available that discuss the research on antifungal nanosystems. Some researchers have summarized the application of nanosystems for the treatment of specific fungal infections [31,32,33]. Additionally, other scholars have introduced different types of nanosystems, such as metal-based, liposomal, and polymer-based systems, for antifungal therapy [34,35]. However, the main objective of these reviews is to introduce different types of nanoparticles, and they do not provide an in-depth summary of the various polymer types used in antifungal nanosystems. While some researchers have provided overviews of nanosystems based on chitosan [36,37], liposomes [38,39], and magnetic [40] nanoparticles for antifungal drug delivery, there is currently no comprehensive review or detailed introduction specifically dedicated to the different polymer types used in constructing antifungal nanosystems.

This review focuses on a comprehensive analysis of polymeric materials commonly used in antifungal drug delivery systems, including chitosan (CS), sodium alginate (SA), gelatin, dextran (Dex), cellulose, and polyester. Although other polymer materials, such as heparin, chitin, and hyaluronic acid, can also be used to design antifungal drug delivery nanosystems, we did not discuss them in this review due to limited research in this area.

Nanoscale fibers formed by electrospinning, mostly used in dressings, are also included in This review, and nanostructures have a larger specific surface area and enable more rapid release at sites such as wounds, mucous membranes, etc. Therefore, we attribute them to antifungal drug delivery nanosystems.

In addition to presenting some interesting and important research works, our review includes a table summarizing antifungal drug delivery nanosystems in recent years, following the introduction of each polymer material. As mentioned earlier, the toxic side effects of current antifungal drugs cannot be ignored, especially for in vivo administration, and antifungal drug delivery nanosystems can help address this issue. The table provides a general overview of how drugs are involved in fungal drug delivery nanosystems, as well as the physical properties (such as the role of polymers, size, potential, mode of binding, release behavior, etc.) of these nanosystems. The “in vivo study” section in the “Administration route/in vivo study” column indicates whether the article includes experimental results from in vivo studies (Y) or not (N). The ‘/’ or ‘N’ in tables indicate that the corresponding data is not mentioned in the paper. We hope that these works will inspire researchers to design nanosystems for fungal drug delivery.

## 2. Polymers for Antifungal Drug Delivery Nanosystems

Polymers have been extensively studied and applied in biomedicine and are generally divided into two categories: degradable and non-degradable. The degradability of certain polymers can allow for different release requirements of drug delivery systems. Nanopolymer systems offer many advantages in antifungal drug delivery, such as the ability to improve drug loading, the ability to chemically bond drugs to functional groups on the polymer surface, and some polymers even possess inherent antifungal properties [41]. Herein, we will introduce the key roles of some representative polymer materials in antifungal drug delivery nanosystems.

### 2.1. Chitosan

CS is a chitin-derived polysaccharide with high biocompatibility and biodegradability [42,43]. Researchers have extensively studied the antibacterial properties of CS due to its amino and positive electrical properties. The antibacterial activity of chitosan and its derivatives may originate from the interaction between positively charged chitosan molecules and negatively charged residues on the surface of fungal cell walls. The methods for preparing CS nanoparticles (CSNPs) include ion crosslinking of low-concentration CS acid solution and tripolyphosphate by ultrasonic and mechanical stirring, 1-(3-Dimethylaminopropyl)-3-ethyl carbodiimide hydrochloride(EDC)/N-Hydroxy succinimide(NHS) crosslinking and redox with metal ions [44]. These methods can be used to prepare antifungal drug delivery nanosystems.

As a drug-releasing nanosystem, CS can deliver a variety of antifungal factors (such as essential oil [45], fluconazole [46], ceftriaxone, imidazolium zinc [47], berberine [48], enzyme, etc.). CS-based nanospheres (CSNPs) can encapsulate poorly water-soluble drugs to improve their solubility. For example, Su Ma et al. encapsulated curcumin (Cur) into CSNPs. Positively charged NPs tend to bind to negatively charged surfaces [49] (such as many biofilms on the surface of microorganisms). Therefore, positively charged CSNPs can deliver Cur to the biofilm and release the drug, thus directly affecting the internal cells. Thus, CSNP-Cur exhibits higher anti-biofilm activity than free Cur and improves its delivery efficiency.

**Figure 1:**
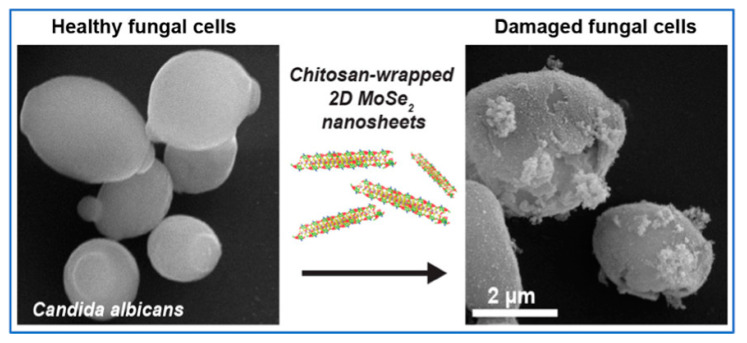
MoSe_2_/Chitosan Nanosheets, Reprinted with permission from [51]. Copyright 2022 American Chemical Society.

Yasser A. et al. [47] obtained a nanosystem encapsulating several essential oils through CSNPs. In addition to delivering imidazolium, this drug delivery nanosystem can also deliver O. syriacum essential oil (OSEO) containing multiple essential oils and a new active complex, Zn (II) Salen. CSPNs significantly enhanced the antibacterial activity of Zn (II) Salen and OSEO. This study demonstrates the applicability of CSNPs as drug-delivery nanosystems for multiple types of drugs. In addition, chitosan exhibits good delivery performance for metal nanoparticles. Biao et al. obtained electrons from silver ions through amino groups in CS to form silver nanoparticles [50]. More interestingly, there are differences in the morphology of Ag-CS produced by the system at different pH values. The reaction system obtained triangular nanosheets at pH = 4.0, while nanoparticles with monodispersity and stability were obtained at pH = 5.0. Recent studies have shown the potential of the morphology of nanosheets in antifungal applications. Sanchari Saha et al. peeled off MoSe_2_/CS nanosheets with synergistic antibacterial effects in the liquid phase [51]. MoSe_2_/CS nanosheets cause fungal cell death through membrane damage, membrane depolarization, metabolic inactivation, and cytoplasmic leakage without requiring more complex modifications and external NIR-assisted photothermal action, as shown in Figure 1. In addition to its excellent antifungal activity, MoSe_2_/CS nanosheets have a high degree of biocompatibility with mammalian cells. The excellent antifungal performance of MoSe_2_/CS nanosheets indicates that they are a promising new type of antifungal agent with potential applications in various biomedical applications. These applications are particularly important given the threat of fungal pathogens.

We present the statistics in Table 1 for the research of chitosan for antifungal drug delivery nanosystems in recent years. The amino groups carried by chitosan endow the antifungal delivery system prepared from chitosan with natural antibacterial effects. Although most studies have shown the great potential of chitosan-based drug carriers, the research on their post-administration effects is still in the exploratory stage. Most studies lack in vivo antifungal results.

### 2.2. Alginate

SA is a widely studied biopolymer with non-toxic, biocompatible, nonimmunogenic, biodegradable, and mucus adhesive properties [52,53,54,55]. SA can be chelated with Ca or other divalent cations to form a gel through the side carboxylic acid part of the G unit. This gel structure is called an ‘egg-box’ structure [56,57]. Therefore, alginate biopolymers can be used to stabilize inorganic metal nanoparticles, and this delivery method is not toxic [58]. That makes SA widely used in antifungal drug delivery nanosystems [57,59].

Abid S et al. prepared SA microspheres with calcium chloride as a crosslinking agent through ion gel technology and used them to coat MgO-CuO nanoparticles loaded with nystatin [60]. Microspheres reduce the specific surface area and reactivity of metal nanoparticles in the human body, providing a safe and improved release mechanism, thereby reducing the toxicity of nanoparticles in direct contact with the human body. At the same time, the nystatin composite loaded microspheres system enables the sustained release of the antifungal agent, which helps to prevent or minimize the occurrence of infection.

**Table 1:** Chitosan for antifungal drug delivery nanosystems.

Loaded Drugs	Role of Chitosan	Other Components	Fungal	Zeta Potential (mV)	Diameters (nm)	Loading Content (LC)	Encapsulation Efficiency (EE)	Drug Release	PDI	Antifungal Efficacy In Vitro	Administration Route/In Vivo Study	Ref.
*O. syriacum* essential oil (OSEO) and imidazolium-Zn(II)Salen	matrix	/	*Candida albicans*	+58.39	120.15 ± 62.65	22.41%	35.17%	80% (50 h)	0.31–0.39	ZOI: 29.48 ± 1.26 mm; MIC; 3.25–45.25 μg/mL	N/N	[47]
Iron oxide nanoparticles/chlorhexidine (CHX)	matrix	/	*Candida albicans/Aspergillus flavus*	+18.10 ± 0.82	33.6 ± 10.7	/	/	/	1.25 ± 0.06	MIC: 400 μg/mL	topical administration/N	[49]
Cinnamic acid grafted CS	matrix	cinnamic acid grafted chitosan	*M. canis*	−69.74	263.0 ± 81.4	/	84.93%	/	/	inhibition: 53.96%, MIC: 200 μg/mL	vaginal administration/N	[45]
Metronidazol	coating layer	/	*C. albicans*	+10.6 ± 1.3	188.7	/	12 μg/mg	63% (8 h)	/	MIC: 18 to 36 μg/mL	N/N	[61]
Fluconazole (Flu)	matrix	/	*C. albicans*	+3.36	82	60.2%	78.7%	8.12% (94 h)	/	MIC: 1.25 mg/mL, ZOI: 22.3 ± 1.6 mm	N/N	[46]
Ceftriaxone	matrix	/	*/*	+32 ± 2.4	56	54.37%	79.43%	8.12% (94 h)	/	ZOI: 19.5 ± 0.6 mm	N/N	[62]
Carvacrol	matrix	/	*C. albicans, C. glabrata, C. krusei*, *C. tropicalis*	/	281.6 ± 2	25.5%	56%	50% (72 h)	0.235 ± 0.03	MIC: 24 μg/mL	N/N	[63]
AmB	matrix	/	*C. albicans*	+15.84 ± 1.41	174.47 ± 5.12	3.05 ± 0.13%	/	80.6% (25 h)	0.17	MIC: 1 μg/mL	N/N	[64]

The ‘/’ or ‘N’ in the table indicates that the corresponding data is not mentioned in the paper, while ‘Y’ indicates that there is relevant data.

**Figure 2:**
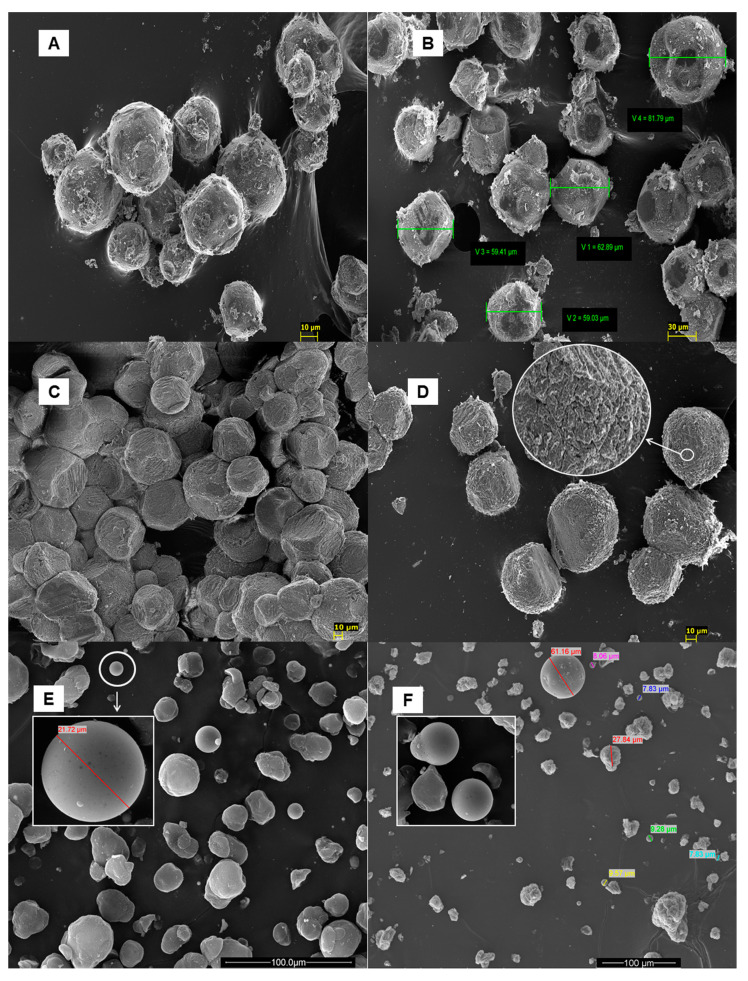
Scanning electron microscope micrographs of the freeze-dried unloaded alginate microspheres (**A**), unloaded CS-coated microspheres (**C**), unloaded hydrogel microspheres (**E**), drug-loaded alginate microspheres (**B**), drug-loaded CS coated microspheres (**D**) and drug-loaded hydrogel microspheres (**F**). Published by Elsevier, 2015 [65].

The cross-linking properties of SA and divalent cations are commonly used to cross-link with Ca^2+^ ions to form microspheres. Microspheres of different sizes can be obtained through ultrasound and water/oil (W/O) emulsification. The microspheres are directly used to encapsulate drugs for delivery and release. These microspheres are used to encapsulate drugs for delivery and release directly. For example, María J. Martín et al. used alginate microspheres as nystatin carriers for oral mucosal drug delivery, enabling the microspheres to come into close contact with the mucosal surface, as shown in Figure 2 [65]. These Nys-loaded microspheres were successfully prepared by emulsification/internal gelation method, showing a significant inhibitory effect on the growth of Candida albicans, indicating its potential clinical use without systemic absorption or tissue damage.

The (1–4)-linked β-d-mannuronic acid (M Unit) and α-l-glucuronic acid (G unit) of SA exhibit anionic properties. They provide mucus penetration for nanoparticles through repulsive interactions with negatively charged sialic acid in the mucosal layer [66]. Vaishnavi et al. coated CS nanospheres with SA, enabling nanoparticles to exhibit better retention efficiency, loading capacity, release kinetics, and corneal permeability [67]. Due to changes in the particle size and surface energy of nanoparticles, they can effectively penetrate the thick mucin layer, which can effectively treat fungal keratitis and deep corneal ulcers.

We present the statistics in Table 2 for the research of alginate for antifungal drug delivery nanosystems in recent years. Sodium alginate has gained much attention in the application of drug delivery as a marine source natural polymer. However, in the construction of nanosystems, current preparation methods mostly use divalent cations to interact with sodium alginate to become nanoparticles. DPI-related data were also not available in some studies. The ion-chelating properties of sodium alginate make it easier to form microspheres. The size of the nanoparticles is not easily controlled, and the size distribution is wide. Researchers may be able to find more efficient ways of dispersion in the future. The abundant hydroxyl groups on the molecular chains of alginate can also serve as an entry point for its modification, thereby endowing alginate with new functions.

### 2.3. Gelatin

Gelatin is a product of incomplete hydrolysis of collagen extracted from animals. However, the hydrolyzed polypeptides have different lengths and usually have a certain width of molecular weight distribution. Gelatin is easily absorbed by the human body due to its hydrolysate being amino acids, resulting in nutritional value. However, gelatin nanomaterials themselves are not antibacterial, and the solubility of gelatin in water is not stable, which is easily affected by temperature. Therefore, gelatin and its modified products are often used as carriers of antibacterial drugs. Compared to synthetic polymer materials, gelatin nanomaterials have lower biological toxicity in terms of antifungal activity. The abundant active groups in gelatin make it easy for nanomaterials to improve their mechanical and rheological properties through crosslinking and chemical modification [72].

In the nanoscale range, gelatin can be made into nanoparticles and nanofibers as carriers of antifungal drugs (such as amp B, Daptomycin [73], Polymyxin B [74], Tobramycin, Vancomycin, etc. [75]).

Hassan M et al. synthesized gelatin nanoparticles by dissolvent method and loaded them with chidamycin and chloramphenicol to improve the antifungal properties of external gauze [76]. The two-step solvent removal method creates gelatin nanoparticles (GNPs) with a low aggregation trend within a limited size range. GNPs contain spectinomycin and chloramphenicol to enhance the treatment of bacterial and fungal infections. The results showed that gelatin nanoparticles loaded with research antibiotics and cellulose cotton gauze treated with these particles exhibited higher antibacterial activity against the bacteria and fungi studied. This is due to the presence of drugs, the safety of nanostructures, and their biocompatibility with skin cells.

V. Aparna et al. innovatively used AutoDock software to calculate and select modified gelatin A nanoparticles to deliver Amp B [77]. Under the action of cross-linking agents, modified gelatin nanoparticles were prepared and delivered to macrophages to treat intracellular fungal infections. Amp B Loaded Gelatin A NPs and Carboxymethylated ι-Carrageenan are combined to achieve the treatment of intracellular Clostridium smooth infection. CMC-Amp B-GNP exhibits appropriate stability, cell compatibility, and blood compatibility.

**Figure 3:**
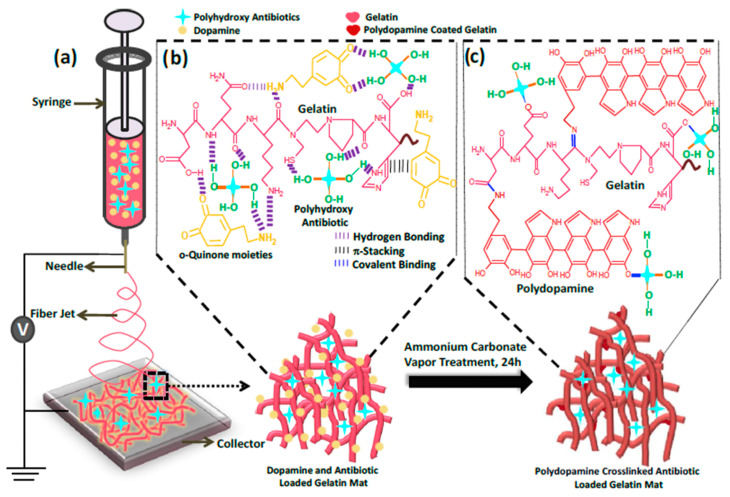
Schematic showing (**a**) Electrospinning set-up used to prepare polydopamine crosslinked polyhydroxy antimicrobials loaded gelatin nanofiber mats. (**b**) Different non-covalent interactions are involved among dopamine, gelatin chains, and polyhydroxy antimicrobials in dopamine and antibiotic-loaded gelatin mats. (**c**) Other covalent interactions involved in polydopamine crosslinked antibiotic-loaded gelatin mats are responsible for the enhanced antimicrobial durability of wound dressings. Published by Elsevier, 2017 [75].

Although gelatin cannot maintain structural stability in an aqueous environment, some researchers have also achieved the preparation of nanoscale fibers using an electrospinning process. Chetna Dhand et al. used drugs rich in hydroxyl groups to improve the water stability of gelatin nanofibers and prepared polydopamine crosslinked gelatin nanofibers as scald wound dressings, as shown in Figure 3 [75]. The method can be extended to impart broad-spectrum antibacterial activity by binding to an antibiotic mixture and retaining long-term antibacterial activity. It was further demonstrated that the electrospun gelatin loaded with vancomycin was directly electrospun onto the bandage gauze, then cross-linked, and its efficacy was examined in an animal model simulating the pathophysiology of human burn wounds. The results confirmed that polydopamine cross-linking did not interfere with wound healing; however, the incorporation of vancomycin enhances wound closure and reduces inflammation. In addition to delivering drugs, researchers have also used polyvinyl alcohol and gelatin blends to improve the properties of gelatin nanofibers [78]. This preparation method makes the selection of gelatin nanofibers for drug delivery more extensive.

We present the statistics in Table 3 for the research of gelatin for antifungal drug delivery nanosystems in recent years. Gelatin consists of a variety of amino acids and has the properties of partial proteins. Since gelatin possesses ampholyte properties can respond in different pH environments. It is commonly used to constitute a drug delivery system with a targeting effect. Due to the presence of polar groups in collagen molecules, nanoparticles with smaller particle sizes are not easily formed, usually all with particle sizes above 100 nm. Gelatin has a relatively short degradation time, and achieving a stable, controlled release system requires modification of collagen; a large number of active groups on the molecule provides great potential for modification. The complex groups of gelatins are more prone to cross-linking and forming a network to achieve drug delivery. However, the interactions between the chains of gelatin molecules are strong, and aggregation between particles is easily formed. Moreover, the rapid degradation of gelatin is not easy to achieve long-term drug release.

### 2.4. Dextran

Dextran is a non-toxic, biocompatible, biodegradable, and hydrophilic natural polysaccharide [81,82]. Dextran can enhance the stability of the drug delivery system and avoid accumulation in blood circulation. Activating macrophages and neutrophils can increase the content of leukocytes, cytokinins, and special antibodies, comprehensively stimulating the immune system of the body. Due to the rich hydroxyl groups in dextran, it can directly bind to biologically active molecules [83,84,85]. In addition, dextran acts as a nanosystem and can form hydrogels [86], films [87], and other systems for drug release. Dextran is easily modified by chemical means and can be derived and modified by etherification, esterification, amidation, and oxidation. The chemical modification rate is as high as 30%, which can also maintain the biodegradability of the skeleton [88,89]. These advantages provide a basis for designing and preparing antifungal drug delivery nanosystems [90,91].

Cristina et al. found in their research that, although dextran itself does not possess antifungal properties, it significantly enhances the stability, magnetic behavior, and biocompatibility of inorganic nanoparticles when coated with dextran on the surface of iron oxide nanoparticles [92]. In addition, by utilizing the drug-loading properties of dextran to load curcumin onto nanoparticles, the antifungal properties of the oxidized nanoparticles were synthesized to enhance the antibacterial activity of cerium dioxide nanoparticles by changing the pH value (i.e., ion balance) of the local environment. In addition, dextran-coated cerium dioxide nanoparticles can enhance the antibacterial activity of nano-ceria by changing the pH value of the local environment (ion balance). In addition to being used as a coating material for nanoparticles, dextran can also be used to synthesize stable silver nanoparticles through chemical reduction and other methods. The reduced silver nanoparticles have good antibacterial properties. Milorad et al. synthesized dextran sulfate stabilized silver nanoparticles (AgNPs-DSS) using a chemical reduction green synthesis method [93]. DS provides structural stability for AgNPs-DSS as a capping agent. Although there are uncertainties in organisms and other factors, AgNPs-DSS have an inhibitory effect on fungi at low concentrations.

**Figure 4:**
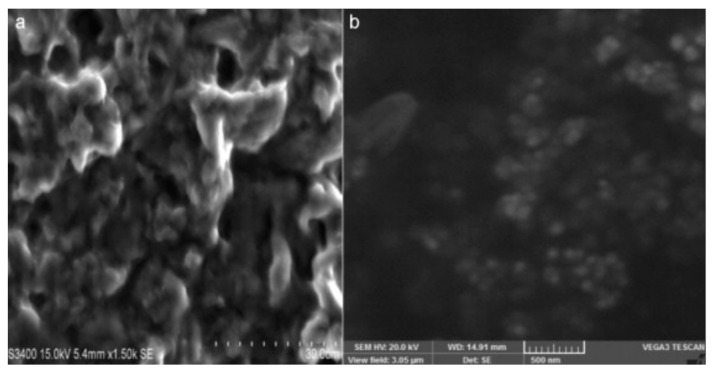
SEM images of β-glucan (**a**) and β-glucan nanoparticle (**b**) Published by Elsevier, 2014. [94].

S. Anusuya et al. obtained β-d-glucan particles in an uncomplicated manner, as shown in Figure 4 [94]. Although this study, in addition to testing against fungi, only β-d-glucan was simply characterized. However, the simple process and uncomplicated composition make it reasonable to believe in the potential application of β-d-glucan particles in biomedicine. Researchers have further enhanced the application potential of dextran through modification. For example, Tuchilus et al. synthesized cationic amphiphilic glucan derivatives with long alkyl groups at the reducing end of the polysaccharide chain [95]. The quaternary ammonium group is connected to the main chain of the main dextran, thereby obtaining a modified dextran with broad-spectrum application potential as an external antifungal agent.

We present the statistics in Table 4 for the research of dextran for antifungal drug delivery nanosystems in recent years. As one of the components of fungal cell walls, glucan has been relatively less studied in the delivery of antifungal drugs. Moreover, nanosystems composed of dextran release rapidly after drug loading and are not suitable for situations where long-term antifungal activity is required. However, as a nontoxic natural macromolecule, glucan is believed to have a positive impact on human immunity. Therefore, there is rich research potential for the application of dextran in antifungal aspects. Researchers can modify the long-term drug-loading properties of dextran through chemical modifications to extend the drug release time.

### 2.5. Cellulose

Cellulose, the major component that makes up the cell walls of plants and algae and part of the microbial capsule [101,102], can be obtained by cellulose extraction and artificial synthesis [103,104]. Cellulose can be prepared into cellulose nanoparticles and nanofibers [105,106]. However, cellulose itself is not anti-fungal, and it is often necessary to synergize it with other antibacterial drugs or antibacterial polymers [107,108,109]. Cellulose units contain three hydroxyl groups. These groups can also be transformed into various functionalities without affecting cellulose structure [107,110]. Cellulose has been widely studied due to its wide source and simplicity of preparation [111,112,113]. Therefore, nano cellulose is widely used in antifungal applications, such as wound dressings and drug carriers [114].

Carla Vilela X et al. combined bacterial nanocellulose (BNC) with monomers of antibacterial polymers (poly[2-(methacryloyloxy)ethyl] trimethyl ammonium chloride, PMETAC) to prepare layered nanofilms for the treatment of fungal infections [115]. These cationic nanocomposite PMETAC/BN materials have UV-blocking properties, high water absorption capacity, thermal stability up to 200 °C, and good mechanical properties. PMETAC/BN has no cytotoxicity to HaCaT cells and can inactivate Candida albicans.

Researchers have extensively studied modified cellulose derivatives. Rimpy et al. prepared fluconazole containing 3D scaffolds by modifying plant-derived nanocellulose with tetraethyl orthosilicate (TEOS) [116]. The swelling, porosity, and tensile strength of TEOS-modified nanocellulose scaffolds were significantly improved. Silica groups added to nanocellulose enhanced mucoadhesive strength, antifungal properties, and ex vivo vaginal penetration ability of lyophilized scaffolds. TEOS-modified nanocellulose scaffolds also exhibited prolonged drug release behavior in SVF buffer up to 24 h, being histologically safe and less cytotoxic to Vero cell lines. Therefore fluconazole loaded TEOS modified cellulose scaffolds have great potential for vaginal drug delivery applications.

**Figure 5:**
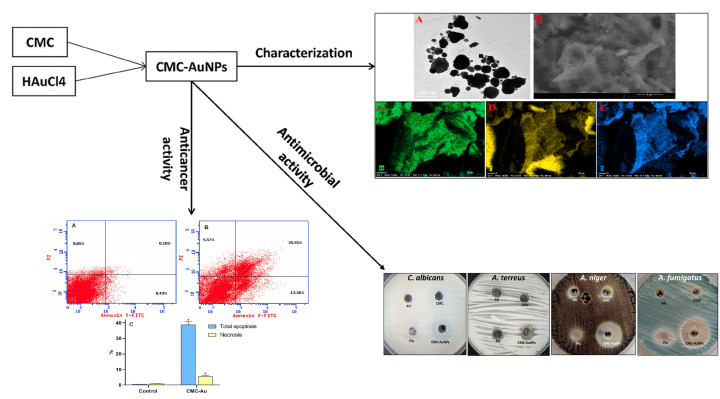
Nanocomposite based on gold nanoparticles and carboxymethyl cellulose. TEM image (right A), SEM image (right B), and SEM/EDX mapping analysis (right C–E) of CMC-AuNPs. CMC-AuNPs induces apoptosis in MCF-7 cells. (left A) Control, (left B) CMC-AuNPs, and (left C) Represent the illustration for % of necrotic and apoptotic cells in different treated cells. Published by Elsevier, 2022 [117].

Ahmed S. et al. prepared a nanocomposite based on gold nanoparticles and carboxymethylcellulose against Aspergillus through a green-friendly chemical reduction method, as shown in Figure 5 [117]. According to cell cycle analysis, CMC AuNPs induced apoptosis and necrosis of liver cancer cells and arrested the cell cycle at G1/G0 phase. Ultimately, the as-prepared nanocomposite CMC AuNPs exhibit good antibacterial, antifungal, and anticancer activities and can be used in pharmaceutical and medical fields. Megha et al. used stabilizing agent hydroxypropyl methylcellulose (HPMC) to limit particle growth during fungal drug griseofulvin (GF) composite synthesis adsorbed on the surface of hydrophilic diatoms [118]. This study provides a unique structure with a diatom onto which hydrophobic drugs can be immobilized to improve drug delivery. The nanoparticle drug release rate after controlling the particle size with HPMC was significantly increased.

We present the statistics in Table 5 for the research of cellulose for antifungal drug delivery nanosystems in recent years. Cellulose has a large molecular weight in natural polysaccharide polymers, and there is a lack of cellulosic enzymes in the human body. Generally, it does not introduce toxicity other than drugs after taking cellulose preparations. On the other hand, cellulose drugs cannot be degraded by the human body if implanted in the body. Therefore, in antifungal applications, nanosystems composed of cellulose are typically only used for surface fungal infections. How to modify cellulose so that it can be degraded and absorbed in vivo is an important research direction.

### 2.6. Polyesters

Polyester materials are the most widely used biodegradable synthetic polymer materials [124,125,126]. Among them, hydroxy acids and lactone polymers represented by polylactic acid (PLA) were first used in the biomedical field and have been certified by the US Food and Drug Administration (FDA) [127]. Polyester-based materials are more hydrophobic in molecular structure and more stable in structure than hydrophilic macromolecules like polysaccharides, giving them unique advantages for applications as drug delivery nanosystems [128,129,130]. The development of electrospinning technology has made up for the shortcomings of polyesters in poor thermal stability during processing [131,132]. Therefore, polyester materials and their copolymers have been widely studied in antifungal applications [133,134,135]. In terms of antifungal applications, polyesters can be applied in the biomedical field as antibacterial drug dressings by preparing nanofibrous membranes using electrospinning technology [136,137,138]. We present the statistics in Table 6 for the research of polyesters for antifungal drug delivery nanosystems in recent years.

**Figure 6:**
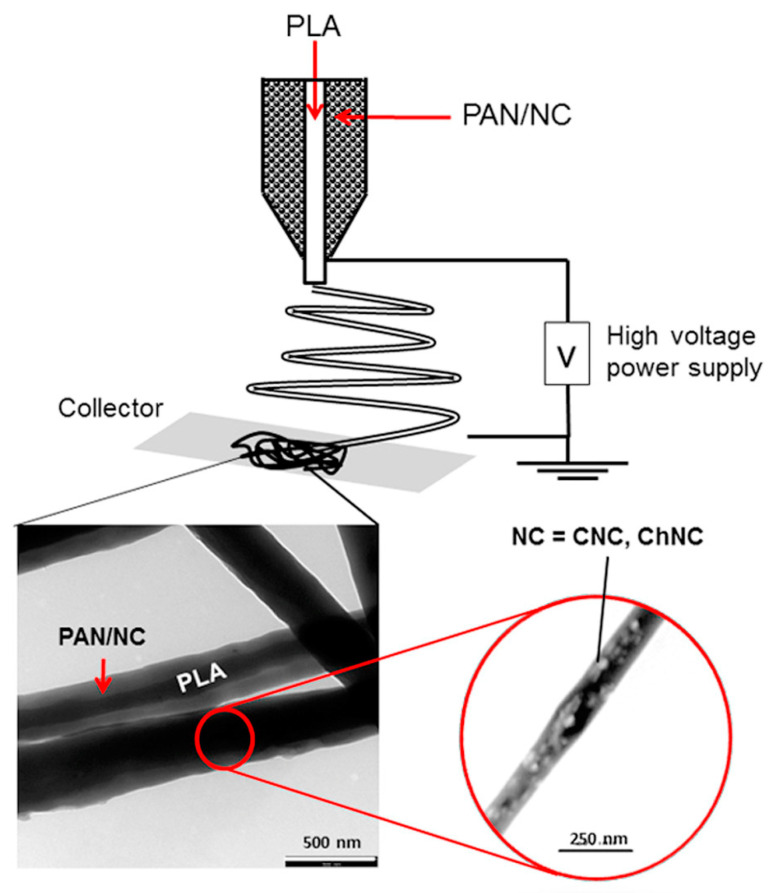
Transmission electron microscopy pictures of core–shell nanofibers prepared by coaxial Electrospinning technology. Published by Elsevier, 2022 [140].

Raul Machado et al. made bovine lactoferrin (bLF) and PLA form uniform, smooth nanofibers (fiber minimum diameter of 380 nm) by electrospinning technique [139]. The final formed nanofibrous membranes had porosity up to 80%. The high porosity and uniform fibers enabled a slow and uniform release of bLF mixed in PLA at 60 days. bLF-PLLA membranes did not induce cytotoxicity in human fibroblasts, and 20 wt% of bLF-PLLA membranes were able to induce cell proliferation even after 24 h of indirect contact. The composite membranes showed very potent antifungal activity against the filamentous fungus A.nidulans. Polylactic acid nanofibers can also be given more possibilities by more complex coaxial electrospinning. B. Jalvo et al. prepared a core–shell nanocomposite membrane with polylactic acid as the core, as shown in Figure 6 [140]. Chitosan on the fiber surface makes the nanofibers positively charged and not prone to microbial colonization. Such that bacteria in contact with the chitosan membrane surface undergo cellular damage.

Nanofibrous membranes can be used for antifungal drug delivery in superficial layers, such as the oral cavity and skin. Copolymers of polylactic acid can also be used for drug delivery by forming nanoparticles that are more widely applied. A novel nanoantibiotic system based on mesoporous silica encapsulated in PLA nanoflowers (PLA-NFs) was developed by Mostafa F. Abdelbar et al. [141]. This mesoporous silicate has a two-dimensional hexagonal porous array and high surface area sensitivity. Such mesoporous silicates exhibit 2D porous hexagonal arrays and high sensitivity of the surface area. The nanoantibiotic system combines polylactic acid nanoflowers with mesoporous silica, which enables the antimicrobial drug (levofloxacin) to be released in a controlled manner under a pH environment. PLA-NFs exhibited a rather fast degradation rate during hydrolysis under an acidic environment, allowing the drug (Levofloxacin; LVX) in the delivery system to be released under controlled conditions.

**Table 6:** Polyesters for antifungal drug delivery nanosystems.

Polymers/Role	Other Components	Loaded Drugs	Fungal	Zeta Potential (mV)	Diameters (nm)	Loading Content (LC)	Encapsulation Efficiency (EE)	Drug Release	PDI	Antifungal Efficacy In Vitro	Administration Route/In Vivo Study	Ref.
PLA/matrix	/	Bovine Lactoferrin	*Aspergillus nidulans*	/	495 ± 127	20 wt%	/	17.7 ± 4.4% (7 weeks)	/	Significantly inhibit mycelium growth	Antifungal dressings/N	[139]
PLA/coating layer	Mesoporous silica nanoparticles	Levofloxacin	*Candida albicans*	/	5.4	33.3 wt%	98.32%	92% (280 min)	/	ZOI: 43 mm at 72 h	N/N	[141]
PLA/core	Polyacrylonitrile/cellulose	Chitin	*Aspergillus niger*	−10.5 ± 1.3	350–400	15 wt%	/	/	/	>99% for fungal spores (>2 µm)	N/N	[140]
PLA/matrix	Cellulose nanofibrils	Silver nanoparticles	*Fusarium/Aspergillus/Curvularia*	/	1.44 ± 0.32 μm	<0.1 wt%	/	/	/	inhibition > 95%	Antifungal dressings/N	[137]
PLGA/matrix	/	Amp B	*Candida albicans*	−10.9 ± 1.9	343.17 ± 24.74	5.7%	85%	45.6% (48 h)	/	inhibition: 99.65%	Topical administration/Y	[133]
PLGA/matrix	/	Amp B	*Candida albicans*	−10.9 ± 1.9	287.8 ± 8.64	5.7 ± 0.12%	85 ± 2.4%	/	85 ± 2.4	diffusion distance: 1.55 ± 0.11 μm	Topical administration/Y	[142]
PLA/matrix	/	Carvacrol	*Candida albicans*		1.54 ± 1.07 μm	28 wt%	/	90% (150 h)	/	inhibition: 92–96%	Antifungal dressings/N	[136]
PLA/matrix	PEG	Amp B	*Candida albicans*	/	25.3 ± 2.7	40 mg/batch	56.5 ± 3.9%	59.4 ± 5.7% (24 h)	/	inhibition: 90.8%	Oral administration/Y	[143]
PLGA/core	/	Butenafine	*Candida albicans, Aspergillus niger*	−20.3	267.21 ± 3.54	1%	72.43 ± 3.11%	42.76 ± 2.87% (48 h)	0.227	ZOI: 20.54 ± 1.8 mm at 48 h	Topical administration/N	[144]
PLA/matrix	Cashew gum	Amp B	*Candida albicans*	−24.3 ± 2.3	1025 ± 143	9.1%	89.7%	52.2 ± 3.9% (168 h)	0.307	MIC: 0.25 μg/mL	Oral administration/N	[145]
PLA/matrix	/	Hypocrellin A	*Candida auris*	/	699	2%	/	/		inhibition: 99.9%	Topical administration/Y	[138]
PCL/matrix	Squalene	Squalene	*Candida albicans*	−48 ± 2.00	254 ± 6.81	30.98 ± 2.20%	86.09 ± 0.28%	85% (4 h)	0.23 ± 3.03	inhibition: 92.47%	Topical administration/Y	[146]
PCL/coating layer	/	Peppermint oil	*Candida albicans/Aspergillus niger*	/	/	/	/	/	/	ZOI: 20.6 mm at 48 h	Antifungal dressings/N	[147]
PCL/coating layer	/	Essential oils	*Candida albicans*	−11 ± 1	200	52 ± 3%	84 ± 6%	/	0.09 ± 0.02	inhibition: 89%	N/N	[148]
PCL/matrix	/	4-Nerolidylcatechol	*Microsporum canis*	−9.30 ± 0.17	143.5 ± 1.36	/	100%	/	0.232 ± 0.00	MIC: 0.625 μg/mL. MFC: 0.625 μg/mL.	Cutaneous administration/Y	[149]
PCL/coating layer	/	Miconazole nitrate	*Candida albicans*	–31.22 ± 2.1	89 ± 3.63	24.1 ± 0.65%	98 ± 5.21%	90% (48 h)	0.35	MIC: 0.75 μg/mL	Cutaneous administration/N	[150]
PCL/matrix	/	Diphenyl diselenide	*Candida albicans*	−10.1 ± 2.21	240 ± 52	5.07 ± 0.14 mg/g	98%	/	0.17 ± 0.08	MIC: 0.5 μg/mL	Cutaneous administration/Y	[151]
PCL/matrix	/	Amp B	*/*	0	183	5 mg/mL	86%	78% (48 h)	0.211	/	N/N	[152]
PCL/matrix	Pluronic	Chloramphenicol	*Candida*	−22.4	123.5	/	98.3%	88% (96 h)	/	MIC: 2 μg/mL	Antifungal dressings/Y	[153]
PCL/coating layer	Polyethyleglicol	Am B	*Albicans/Glabrata/Auris*	−8.8 ± 0.1	226	16.40 ± 0.18 wt%	/	38% (100 h)	0.25	MIC: 0.11 μg/mL	N/N	[154]

The ‘/’ or ‘N’ in the table indicates that the corresponding data is not mentioned in the paper, while ‘Y’ indicates that there is relevant data.

Due to the limitations of polylactic acid in mechanical properties, current research on polylactic acid nanoscale antifungal is mostly synergistic with other polymers. Polylactic acid glycolic acid (PLGA) exhibits excellent drug loading and antifungal properties. Researchers have characterized the drug-loading properties of PLGA nanoparticles for antifungal drugs, such as butenolone (BT) and Amp B [133,142,144]. In addition, Some researchers explored the enhancement of oral absorption of Amp B by PLGA-PEG nanoparticles [143]. Due to the excellent degradation properties of PLA and PLGA, they are metabolized in the human body within a few weeks and are likely not to cause significant environmental residues [155].

Besides PLA, PCL has also been used by researchers to design antifungal drug delivery nanosystems. Vanessa et al. loaded 4-Neroliyl chloride methanol (4-NC) with PCL, which showed high encapsulation efficiency (100%) for 4-NC [149]. PCL nanoparticles, while retaining 4-NC antifungal activity, also reduced cytotoxicity, increased the stability and solubility of the substance, and increased the efficacy of 4-NC.

Compared with liposomes, PCL has a slower and more stable release behavior. Prepared Mn-loaded PCL nanocapsules with a simple, cost-effective technique by R. S. Abdel-Rashid et al. [150]. The resulting nanocapsules represent a good route to deliver MNS due to their small particle size, slow biphasic release rate, high % EE and high stability. In addition to the above advantages, the nanocapsules have good antifungal activity, dual effects on superficial and deep fungal infections, and biphasic release mode. Therefore, PCL nanocapsules can enhance antifungal activity, minimize side effects, and reduce dosage and administration frequency.

**Figure 7:**
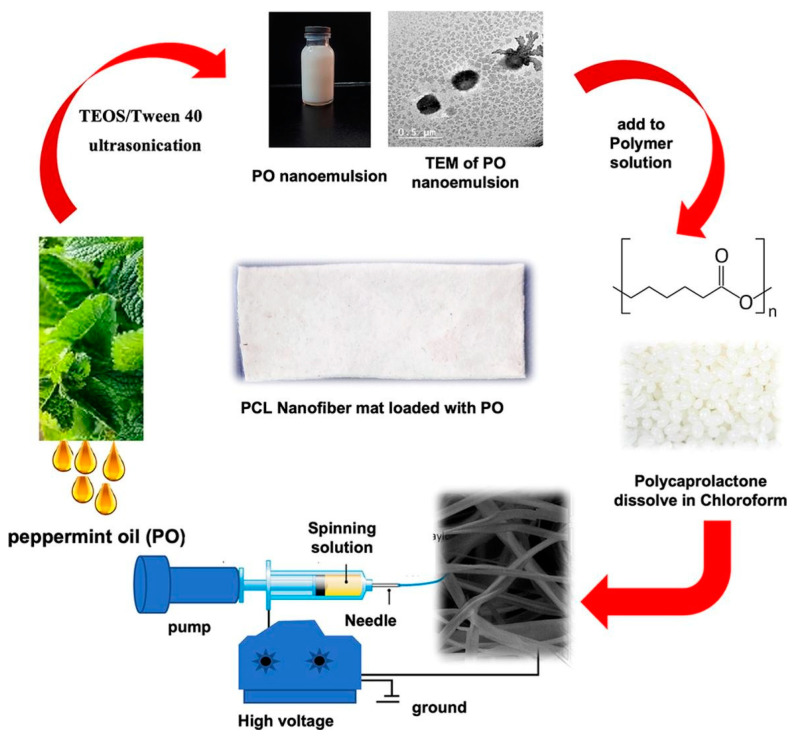
Potential antimicrobial and antibiofilm efficacy of essential oil nanoemulsion loaded polycaprolactone nanofibrous dermal patches Published by Elsevier, 2023 [147].

PCL would also be used by researchers for electrospinning. Mehrez E. et al. blended PCL and peppermint oil (PO) nanoemulsion to prepare uniform nano-sized PO nanoemulsion, as shown in Figure 7 [147]. The electrospinning technique was used to prepare PCL nanofibrous mats loaded with PO nanoemulsion. This nanofibrous mat has good and significant antibacterial and antifungal activity against a variety of human pathogens. The absolute inhibition of biofilm formation was enhanced for the successful encapsulation of the highest concentration of PO nanoemulsion. Because nanofibrous mats are coated with smooth nanofibrous morphology, as well as strong antibacterial activity, they can play a role in superficial antifungal therapy, such as acne and skin diseases and wound healing.

Polysaccharide-based antifungal drug delivery nanosystems often have higher release rates, and it is often difficult to achieve a longer cycle of drug delivery behavior (weeks). While polyester materials do not have a rich range of modified groups, their stability provides unique advantages when drug delivery systems require slower and more stable delivery behavior.

Compared to conventional drug delivery systems, nanosystems exhibit significantly high specific surface areas, which are advantageous for drug loading and release. The antifungal nanosystems based on polymer matrices discussed in this article can be primarily classified into two categories: nanoparticles and nanofibers.

Nanoparticles can be classified into two categories based on their morphological characteristics: nanocapsules and nanospheres. Nanocapsules are composed of a core, which can be oil-based or water-based, encapsulated by a polymer shell. This dual-layer structure allows for the drug to be dissolved in the internal oil-based or water-based phase of the capsule, facilitating drug loading and release. The polymer shell provides protection for the internal drug while maintaining the nanosize of the system, preventing aggregation and fusion of the internal nanophase from forming larger particles. Additionally, the polymer shell can control the release rate of the drug, reducing drug inactivation. On the other hand, nanospheres are composed of a continuous polymer network and can retain the drug internally or adsorb it on the surface through physical adsorption and chemical interactions. Due to their larger specific surface area, nanospheres provide more contact between the drug and the release environment, enabling drug loading and release. Nanocapsules offer controlled release capabilities, while nanospheres, due to their internal continuous polymer network, exhibit higher contact areas for drug interaction.

Compared to nanoparticles, nanofibers exhibit unique characteristics for drug delivery. Nanoparticles are typically employed as drug carriers and require a combination with liquid dispersion systems or solid carriers, such as incorporation into dressings, injectables, or gels, for application. In contrast, nanofibers can directly form fibrous membranes, providing both mechanical strength and drug-loading capability. Due to their porous structure, nanofibers can be utilized as the surface layer of antifungal dressings while maintaining breathability. The development of electrospinning techniques has enabled researchers to fabricate nanofibers using coaxial electrospinning, wherein different drugs are loaded in the core and shell layers to achieve sequential drug release. Furthermore, precise control over the diameter and morphology of nanofibers can be achieved by adjusting process parameters during electrospinning, enabling accurate modulation of drug delivery. Nanoparticle size control is relatively challenging, particularly when compared to nanofiber diameter.

## 3. Summary and Conclusions

In our review of research on polymers, we found that the inherent antifungal properties of most polymers are not ideal. While cationic polymers have a broad range of antibacterial properties, their effectiveness against fungal infections is limited. Therefore, researchers often utilize polymers as carriers for the efficient delivery of antibacterial drugs, constructing antifungal agent delivery nanosystems to combat fungal infections. This approach improves the bioavailability of antifungal drugs, prevents excessive administration due to low absorption efficiency, and reduces the drug metabolism burden on patients. Moreover, the sustained-release delivery nanosystems composed of polymers exhibit high stability, minimizing the toxicity of antifungal drugs and reducing adverse reactions during treatment. By manipulating the length of the molecular chains, the degradation of the polymer can be slowed down in vivo or on the body surface. Loading antifungal drugs onto these polymer systems enables sustained drug release, maintaining a stable drug concentration and prolonging the drug’s efficacy. We also consider scaffolds prepared using nanofibers as a type of antifungal nano drug delivery system. Polymer scaffolds are commonly employed as dressings for treating superficial fungal infections. Researchers have utilized electrospinning technology to transform long-chain polymers into nanofibers, which possess strong adhesion capabilities due to their high specific surface area and surface charge density. This improves their binding with fungi and enhances drug delivery efficiency. Electrospinning allows for the incorporation of other macromolecules or small molecules into the polymer, ensuring more uniform drug loading and release and enhancing the antibacterial effect.

Given the complex and diverse types and sites of fungal infections, different factors must be taken into account when treating infections in vivo or on the body surface. Therefore, the use of antifungal drugs presents numerous complex challenges in practical applications. The diversity of polymers provides us with a range of solutions for addressing these challenges. Whether selecting polysaccharides, protein, or polyesters-based polymers as nano-drug delivery carriers, the ultimate goal is to achieve improved therapeutic effects with fewer side effects in antifungal therapy. When designing antifungal agent delivery nanosystems using polymers, researchers should carefully choose suitable carriers based on the specific scenarios they encounter. In this selection process, we hope that this review can offer valuable guidance and assistance.

## Figures and Tables

**Table 2:** Alginate for antifungal drug delivery nanosystems.

Loaded Drugs	Role of Alginate	Other Components	Fungal	Zeta Potential (mV)	Diameters (nm)	Loading Content (LC)	Encapsulation Efficiency (EE)	Drug Release	PDI	Antifungal Efficacy In Vitro	Administration Route/In Vivo Study	Ref.
Nystatin (Nys)	internal phase	/	*C. albicans*	−37.42 ± 1.07 (pH 7.5); −35.22 ± 1.40 (pH 5.5)	24,410	Surface 7.63 ± 1.81%	/	About 62% (18 h)	/	exhibited a marked fungicidal activity	oral mucosa administration/Y	[65]
						Inside 17.45 ± 2.34%						
Voriconazole	coating layer	Chitosan	*/*	−24 ± 0.9	185 ± 1	10.38 ± 0.87%	91.31 ± 1.05%	About 68% (50 h)	/	/	corneal administration/N	[67]
Miltefosine	matrix	/	*C. albicans*	−39.7 ± 5.2	279.1 ± 56.7	/	81.70 ± 6.64%	55.24% (181 h)	/	MIC: 0.03 to 2 µg/mL	mucosal and oral administration/Y	[68]
			*/C. gattii.*									
Sodium selenate	coating layer	/	*Fusarium oxysporum Schltdl*	−7.25	80	/	/	About 60% (40 h)	/	/	N/N	[69]
Miltefosine	matrix	/	*Galleria mellonella caterpillars*	−39.7 ± 5.2	279.1 ± 56.7	About 80%	81.70% ± 6.64	55.24% (181 h)	/	MIC: 0.03 µg/mL	mucosal and oral administration/Y	[68]
Ketoconazole	matrix	poloxamer 407, carbopol 940	*Candida albicans*	+82.2 ± 64.94	34.8 ± 73.34	/	97.5 ± 41.95%	43.75 ± 5.38% (6 h)	/	/	ocular administration/Y	[70]
Ethionamide	matrix	Chitosan	*Mycobacterial*	−24 ± 9	324 ± 62	59%		About 100% (80 h)	0.35 ± 0.09	MIC: 0.43 µg/mL	inhalation and intravenous administration/N	[71]

The ‘/’ or ‘N’ in the table indicates that the corresponding data is not mentioned in the paper, while ‘Y’ indicates that there is relevant data.

**Table 3:** Gelatin for antifungal drug delivery nanosystems.

Loaded Drugs	Role of Gelatin	Other Components	Fungal	Zeta Potential (mV)	Diameters (nm)	Loading Content (LC)	Encapsulation Efficiency (EE)	Drug Release	PDI	Antifungal Efficacy In Vitro	Administration Route/In Vivo Study	Ref.
Spectinomycin	matrix	/	*/*	/	250.9	0.1–0.5 g/100 mL	/	/	/	ZOI: 22 mm	oral administration/N	[76]
Fluconazole/Cinnamaldehyde	matrix	Poly(Vinyl Alcohol)	*Candida albicans*	/	334 ± 56	0.2 + 2.6 wt%	73.84% (CA) and 68.58% (FLU)	CA87% (8 h)/FLU61% (12 h)	/	ZOI: 36 ± 1 mm	corneal administration/N	[78]
Amp B	matrix	Carboxymethyl ι-carrageenan	*Candida glabrata*	−25 ± 5.3	343 ± 12	2 wt%	78 ± 0.68%	99% (40 days)	<0.3	No viable C. glabrata was detected in Macrophage cells.	N/N	[77]
Amp B	shell-forming components	polyethylene oxide	*Candida tropicalis/Candida krusei/Candida parapsilosis/Candida glabrata/Candida dubliniensis/Aspergillus flavus*	/	351 ± 73	0–9%	/	78% (11 h)	/	ZOI: 19 ± 0.5 mm	Topical administration/N	[79]
Mmethylene blue	matrix	/	*Candida albicans*	30.8	100	3.13% to 6.75%	84.0 ± 1.3%	48% (180 h)	0.107	/	N/N	[80]
Daptomycin/Polymyxin B/Tobramycin/Vancomycin/Caspofungin/Amp B	matrix	polydopamine	*Candida albicans*	/	998 ± 250	0.5%	/	80% (24 h)	/	ZOI: 31 mm	wound dressings/Y	[75]

The ‘/’ or ‘N’ in the table indicates that the corresponding data is not mentioned in the paper, while ‘Y’ indicates that there is relevant data.

**Table 4:** Dextran for antifungal drug delivery nanosystems.

Loaded Drugs	Role of Dextran	Other Components	Fungal	Zeta Potential (mV)	Diameters (nm)	Loading Content (LC)	Encapsulation Efficiency (EE)	Drug Release	PDI	Antifungal Efficacy In Vitro	Administration Route/In Vivo Study	Ref.
Tobramycin/AgNPs	matrix	/	Pseudomonas aeruginosa (PA)	−39.2 ± 1.5	167.2 ± 3.56	>75%	Ag > 95%/Tob78 ± 2.5%	/	0.241 ± 0.008	MIC: 2 μg/mL	intratracheal instillation/Y	[96]
Ciprofloxacin (CIP)/mucolytic enzyme papain (PAP)	matrix	/	PA	−51.0 ± 1.9	223 ± 99	/	88%	100% (40 min)	0.51 ± 0.05	/	N/N	[97]
Curcumin (CUR)	matrix	poly-lactic acid	*/*	+35 (±7.23)	248 (±86.39)	/	73.81%	50% (16 h)	0.21 ± 0.09	/	oral mucosa administration/Y	[98]
Amp B	coating layer	poly-lactic acid	*/*	37	644 ± 52	/	56%	100% (5 min)	0.27	/	intravenous administration/Y	[99]
Itraconazole (ITZ)	matrix	/	*/*	−47 ± 0.8	400 ± 120	65 ± 6%	93 ± 2%	/	/	/	N/N	[100]

The ‘/’ or ‘N’ in the table indicates that the corresponding data is not mentioned in the paper, while ‘Y’ indicates that there is relevant data.

**Table 5:** Cellulose for antifungal drug delivery nanosystems.

Loaded Drugs	Role of Cellulose	Other Components	Fungal	Zeta Potential (mV)	Diameters (nm)	Loading Content (LC)	Encapsulation Efficiency (EE)	Drug Release	PDI	Antifungal Efficacy In Vitro	Administration Route/In Vivo Study	Ref.
[2-(methacryloyloxy)ethyl] trimethylammonium chloride solution	matrix	/	*Candida albicans*	/	/	40%	/	/	/	inhibition > 99.9%	Antifungal dressings/N	[115]
Fluconazole	matrix	tetraethyl orthosilicate	*Candida albicans*	RNF-25.4 ± 1.13/WNF-24.4 ± 1.15	RNF for 441.7/WNF for 407.7	1% *w*/*v*	/	30% (24 h)	0.735 for RNF/0.655 for WNF	ZOI: 39 mm	Vaginal administration/Y	[116]
ciclopirox olamine and Boswellia serrata	matrix	/	*Candida albicans*, *Candida parapsilosis*	/	/	10.1 ± 3.1%	10.0 ± 2.2%	79.1 ± 17.7% (48 h)	/	ZOI: 20 mm	Topical administration/N	[119]
gold nanoparticles	matrix	/	*C. albicans, A. terreus*, *A. niger*, *and A. fumigatus*	−3.16	54.49	/	/	pH 5.5, >45%. pH 7 < 5% pH 9 <1%.	/	MIC: 20 μg/mL	N/N	[117]
hydroxyapatite	matrix	lysine	*Candida albicans*	/	600	50–70%	/	/	/	ZOI: 28 mm	N/N	[120]
Griseofulvin	stabilizer	diatom	*/*	−13 ± 2	2–3 ± 0.5 μm	/	/	/	0.675	/	N/N	[118]
Amp	matrix	/	*Candida albicans*	−16.10 ± 2.6	150 ± 9.23	5 μg/mL	60 ± 2%	18 ± 2.1% (12 h)	0.258 ± 0.005	MIC: 0.145 ± 0.01 µg/mL	Oral administration/Y	[121]
Lliconazole	matrix	Polyvinyl alcohol	*Candida albicans*, *Aspergillus niger*	−14.6–32.3	300–600	1%	70–80%	up to 8 h	0.108~0.497	strong antifungal activity	Topical administration/N	[122]
Citin’ nanocrystals	matrix	/	*Aspergillus*	/	60	0–10 %	/	/	/	inhibition: 98.87%	Antifungal dressings/N	[123]

The ‘/’ or ‘N’ in the table indicates that the corresponding data is not mentioned in the paper, while ‘Y’ indicates that there is relevant data.

## Data Availability

Not applicable.
